# Supplementary material for: Exogenous Sugar Alleviates Salt Stress in Cucumber Seedlings by Regulating the Antioxidant System and Hormone Signaling
Source: Curr Issues Mol Biol. 2025 Sep 12;47(9):754. doi: 10.3390/cimb47090754 (PMC12468133; doi:10.3390/cimb47090754)
Supplement: Supplementary file 1 [file cimb-47-00754-s001.zip › Figure/Figure.pdf]

Figure 1 The growth phenotypes of cucumber seedlings under salt stress with exogenous sugars.

Note: After different pretreatments, the seedlings were subjected to salt stress (150 mmol·L^{-1} NaCl) or non-salt stress for 6 days. CK: Control; S: Salt treatment; G: Pretreated with 0.5 mmol·L^{-1} Glc; T: Pretreated with 0.5 mmol·L^{-1} Suc; G+S: Salt treatment after pretreatment with 0.5 mmol·L^{-1} Glc; T+S: Salt treatment after pretreatment with 0.5 mmol·L^{-1} Suc. A: Jinyou 1; B: Xintaimici.

Figure 2 The detection of chlorophyll and malondialdehyde contents in cucumber leaves under salt stress with exogenous sugar. Note: Note: Values are means ± SD, n = 3; different letters indicate significant differences ($P < 0.05$)

Figure 3 Effects of exogenous sugar on the contents of SOD, POD, CAT and APX in leaves and roots of cucumber seedlings under salt stress. Note: Values are means ± SD (n = 3); different letters indicate significant differences ($P < 0.05$).

Figure 4 Effects of exogenous sugar on the content of soluble sugar and soluble protein in leaves and roots of cucumber seedlings under salt stress. Note: Values are means ± SD, n = 3; different letters indicate significant differences ($P < 0.05$).

Figure 5: **Correlation analysis matrix for each indicator. Note: X-for Xintaimici.** Root length for X-RL; stem length for X-SL; fresh weight of leaves for X-LFW; fresh weight of roots for X-RFW; dry weight of leaves for X-LDW; dry weight of roots for X-RDW; soluble protein for X-SP; soluble sugar for X-SS; relative chlorophyll content for X-SPAD; malondialdehyde for X-MDA; peroxidase for X-POD; catalase for X-CAT; superoxide dismutase for X-SOD; ascorbate peroxidase for X-APX. J-for Jinyou1. Root length for J-RL; stem length for J-SL; fresh weight of leaves for J-LFW; fresh weight of roots for J-RFW; dry weight of leaves for J-LDW; dry weight of roots for J-RDW; soluble protein for J-SP; soluble sugar for J-SS; relative chlorophyll content for J-SPAD; malondialdehyde for J-MDA; peroxidase for J-POD; catalase for J-CAT; superoxide dismutase for J-SOD; ascorbate peroxidase for J-APX. $p \le 0.05$, ** $p \le 0.01$, *** $p \le 0.001$. Red tones indicate positive correlations (positive correlation coefficients ranging from light red to dark red, corresponding to correlation coefficients from 0 to 1, with darker shades representing stronger positive correlations). Blue tones indicate negative correlations (negative correlation coefficients ranging from light blue to dark blue, corresponding to correlation coefficients from 0 to -1, with darker shades representing stronger negative correlations). White represents the absence of a statistically significant correlation (correlation coefficient close to 0 or non-significant correlation).

Figure 6 Transcriptional variation of cucumber plants under different treatments.

Volcano plots analysis of the DEGs in different treatments. (B) Venn diagrams showed the proportions of the up- and down-regulated genes in three treatments. (C) The number of up- and down-regulated genes in different treatments.

Figure 7 Enrichment analysis of transcriptional variation in cucumber plants under different treatments（GO enrichment and KEGG analysis）.

Figure8 Distribution of transcription factor gene families expressed in different comparison group.

Figure 9 Effect of exogenous sugar on the expression of genes involved in hormone metabolism in cucumber seedlings under salt stress. (A) Effect of exogenous sugar on the expression of AUX-related genes under salt stress. (B) Effect of exogenous sugar on the expression of ET-related genes under salt stress. (C) Effect of exogenous sugar on the expression of ABA-related genes under salt stress.

Figure 10 Effect of exogenous sugar on the expression of antioxidant enzyme-related genes under salt stress.

Figure 11 Schematic representation of the strategy of exogenous sugar to alleviate salt stress in cucumber seedlings.
