# Supplementary material for: Exogenous Sugar Alleviates Salt Stress in Cucumber Seedlings by Regulating the Antioxidant System and Hormone Signaling
Source: Curr Issues Mol Biol. 2025 Sep 12;47(9):754. doi: 10.3390/cimb47090754 (PMC12468133; doi:10.3390/cimb47090754)
Supplement: Supplementary file 1 [file cimb-47-00754-s001.zip › Figure/Table.pdf]

Table 1 Effects of Exogenous Sugars on Growth Phenotypes of Jinyou 1 Cucumber Seedlings under Salt Stress

Notes：Values ale meaBs 4-SE (n=3). Letters indicate significant differences at *P*<0.05 according to Duncan'S multiple range tests.

Species	concentration （mmol/L）	Root length （cm/plant）	Stem length （cm/plant）	Fresh weight of leaves（g/plant）	Fresh weight of roots（g/plant）	Dry weight of leaves （g/plant）	Dry weight of roots （g/plant）
Jinyou 1	CK	20.43±1.4a	5.16±0.05bc	10.29±0.27a	1.08±0.21ab	0.82±0.05b	0.05±0.01ab
	S	11.5±1c	5.09±0.08c	6.17±0.05c	0.4±0.07d	0.47±0.03d	0.02±0.01c
	T	25.77±0.93bc	5.59±0.09b	10.66±0.31a	1.25±0.14a	0.92±0.08a	0.06±0.01a
	G	31.5±1bc	8.1±0.53a	10.97±0.64a	1.31±0.11a	1.01±0.06a	0.06±0.01a
	G+S	15.43±1.29b	5.13±0.15bc	7.98±0.29b	0.88±0.05bc	0.68±0.02c	0.04±0.01bc
	T+S	15.67±0.91bc	5.2±0.1bc	8.07±0.28b	0.75±0.04c	0.66±0.03c	0.03±0.01c

Table 2 Effects of Exogenous Sugars on Growth Phenotypes of Xintaimici Cucumber Seedlings under Salt Stress

Notes：Values ale meaBs 4-SE (n=3). Letters indicate significant differences at *P*<0.05 according to Duncan'S multiple range tests.

Species	concentration （mmol/L）	Root length （cm/plant）	Stem length （cm/plant）	Fresh weight of leaves（g/plant）	Fresh weight of roots（g/plant）	Dry weight of leaves （g/plant）	Dry weight of roots （g/plant）
Xintaimici	CK	19.5±1b	6.63±0.47b	8.02±0.42b	0.94±0.14ab	0.89±0.05b	0.08±0.01bc
	S	11.19±1.01d	4.93±0.15c	5.34±0.21c	0.53±0.03c	0.55±0.04d	0.03±0.01e
	T	21.5±1ab	7.69±0.51a	9.97±0.42a	1.04±0.16ab	1.26±0.02a	0.09±0.01b
	G	22.5±1a	6.9±0.46b	10.39±0.52a	1.15±0.27a	1.16±0.1a	0.11±0.02a
	G+S	15.2±1c	5.33±0.42c	6.09±0.43c	0.78±0.08bc	0.69±0.03c	0.05±0.01d
	T+S	14.1±1c	5.7±0.1c	5.73±0.33c	0.77±0.09bc	0.67±0.03c	0.06±0.01cd

Table 3 Summary of Sequencing Data for Cucumber Leaves Samples

Sample	Trimmed_Read_Numbe	Trimmed_Bases	Useful_read%	Useful_bases%
CK1	41801562	6301175388	98.78	98.61
CK2	38540682	5809769571	98.67	98.5
CK3	40756362	6144984555	98.56	98.42
Glu1	40849056	6157622624	98.57	98.4
Glu2	44114928	6643346865	98.66	98.39
Glu3	39828620	5996395865	98.68	98.39
GluSalt1	39724888	5986386299	98.71	98.51
GluSalt2	45010458	6776217132	98.55	98.25
GluSalt3	42776240	6444662183	98.63	98.4
Salt1	45333936	6823398963	98.88	98.56
Salt2	41571160	6259915355	98.72	98.45
Salt3	45697498	6889453658	98.66	98.5
Summary	758,448,556	76,233,328,458		
