# Supplementary figures and images for: Exogenous Sugar Alleviates Salt Stress in Cucumber Seedlings by Regulating the Antioxidant System and Hormone Signaling
Source: Curr Issues Mol Biol. 2025 Sep 12;47(9):754. doi: 10.3390/cimb47090754 (PMC12468133; doi:10.3390/cimb47090754)

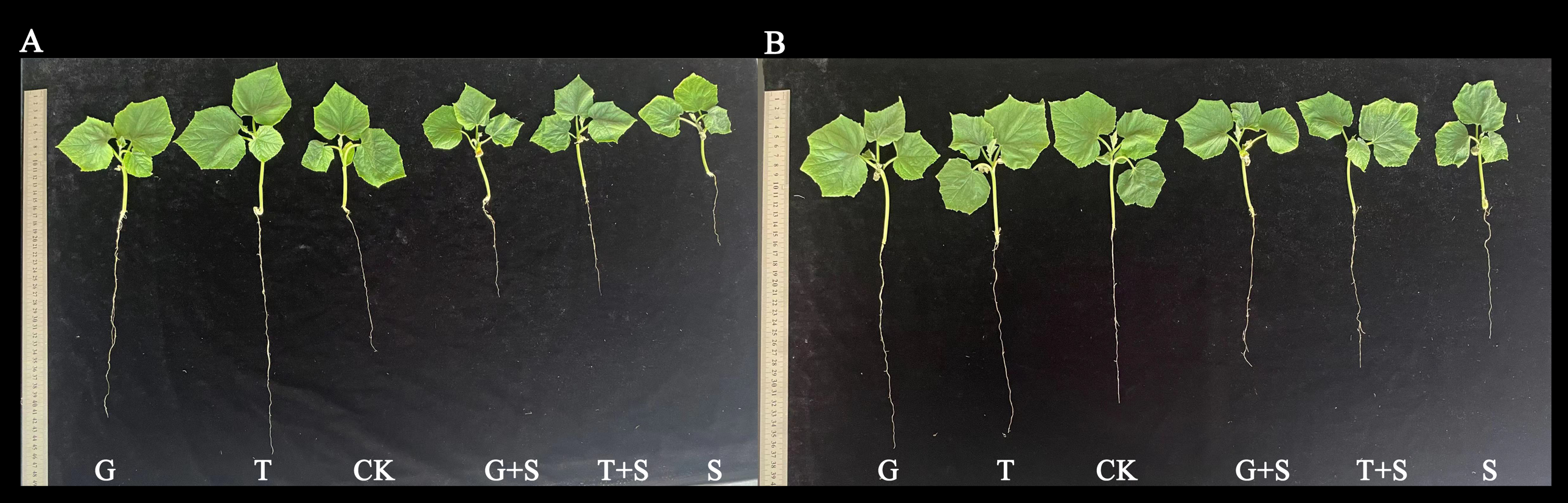

Supplement: Supplementary file 1 [file cimb-47-00754-s001.zip › Figure/Figure 1.tif]

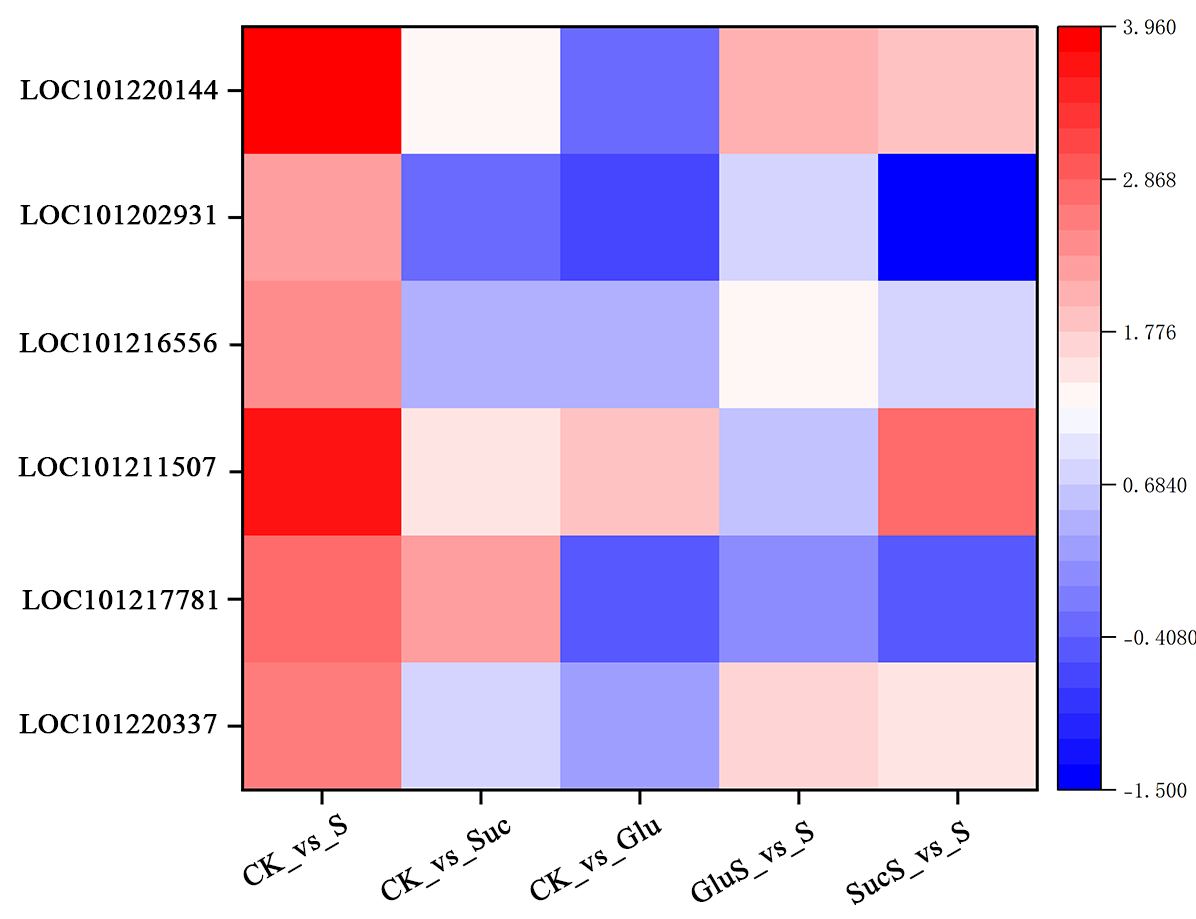

Supplement: Supplementary file 1 [file cimb-47-00754-s001.zip › Figure/Figure 10.tif]

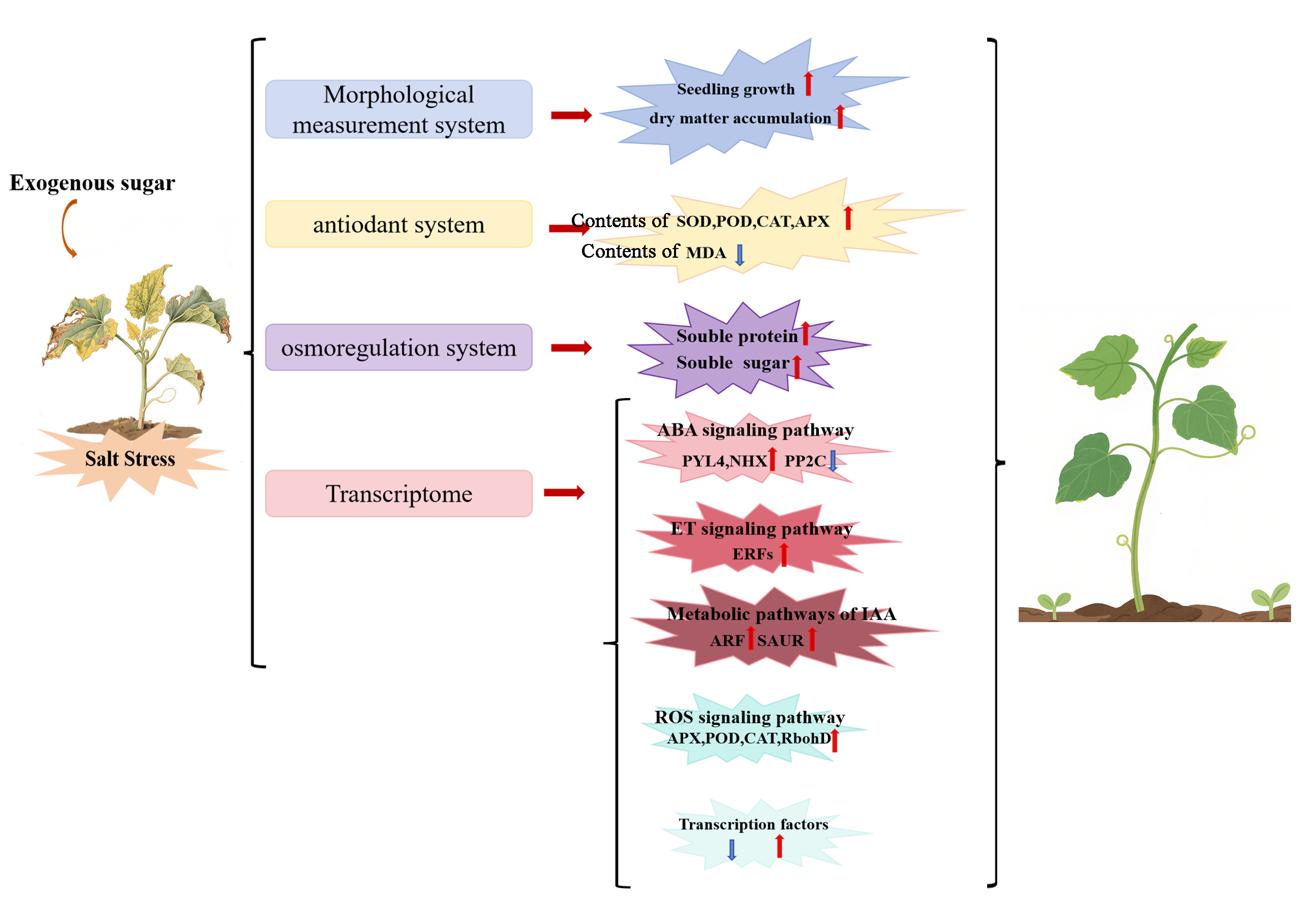

Supplement: Supplementary file 1 [file cimb-47-00754-s001.zip › Figure/Figure 11.tif]

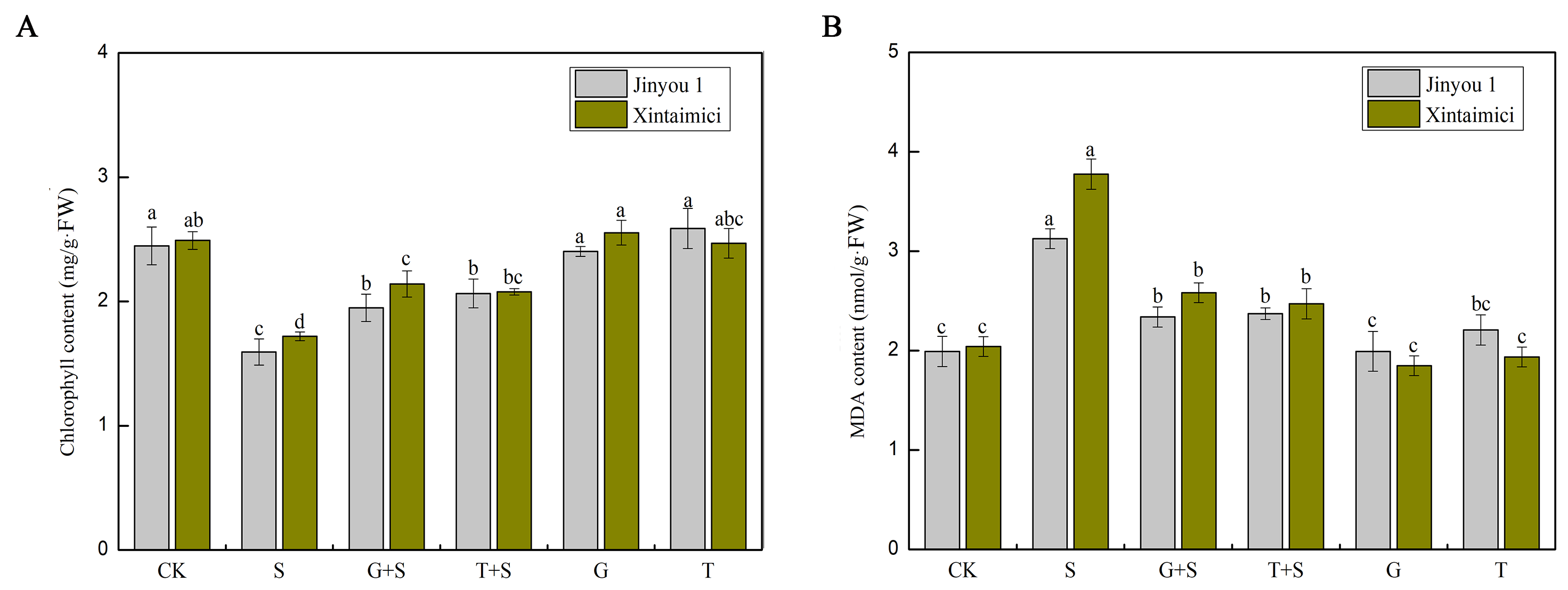

Supplement: Supplementary file 1 [file cimb-47-00754-s001.zip › Figure/Figure 2.tif]

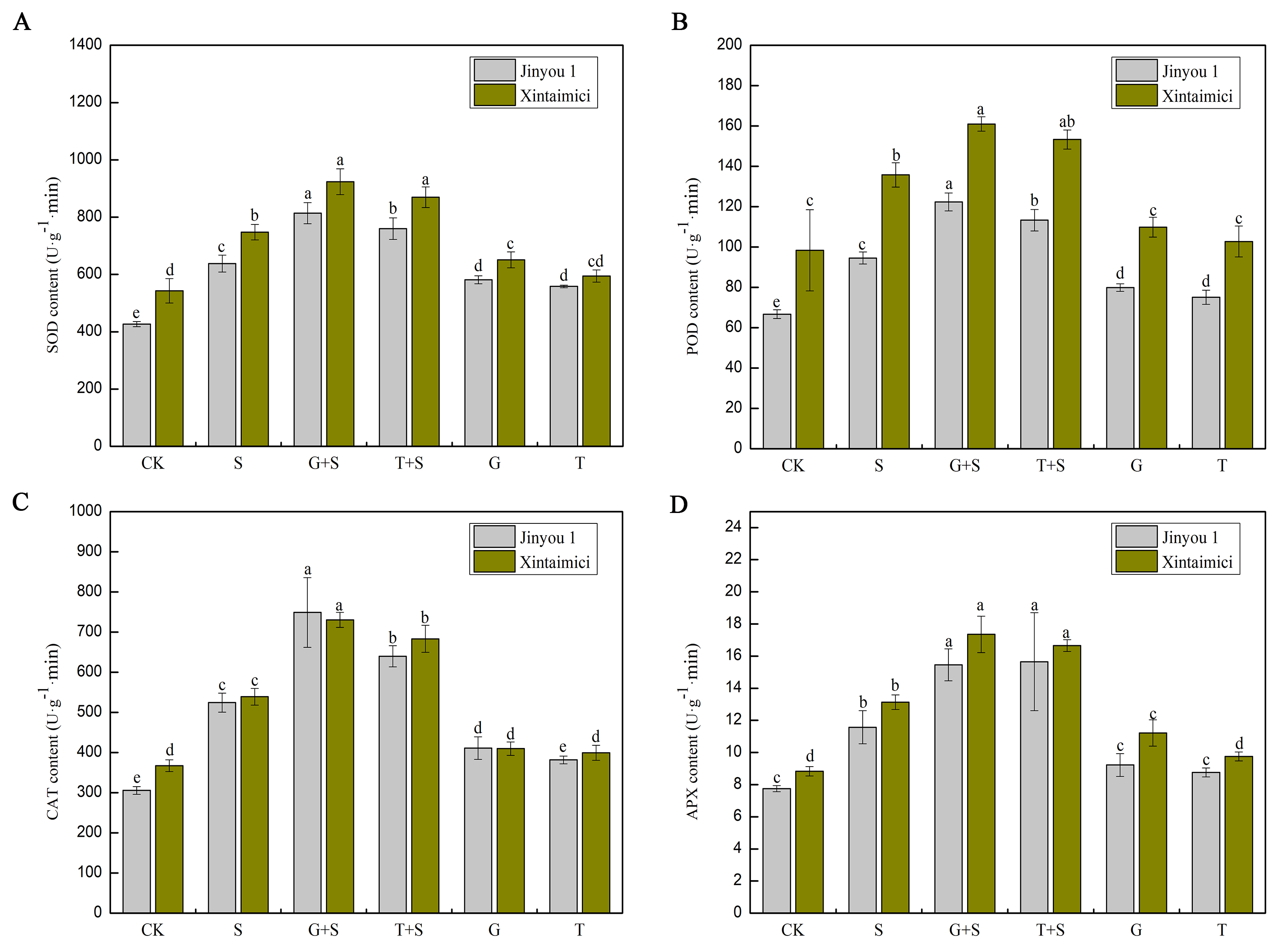

Supplement: Supplementary file 1 [file cimb-47-00754-s001.zip › Figure/Figure 3.tif]

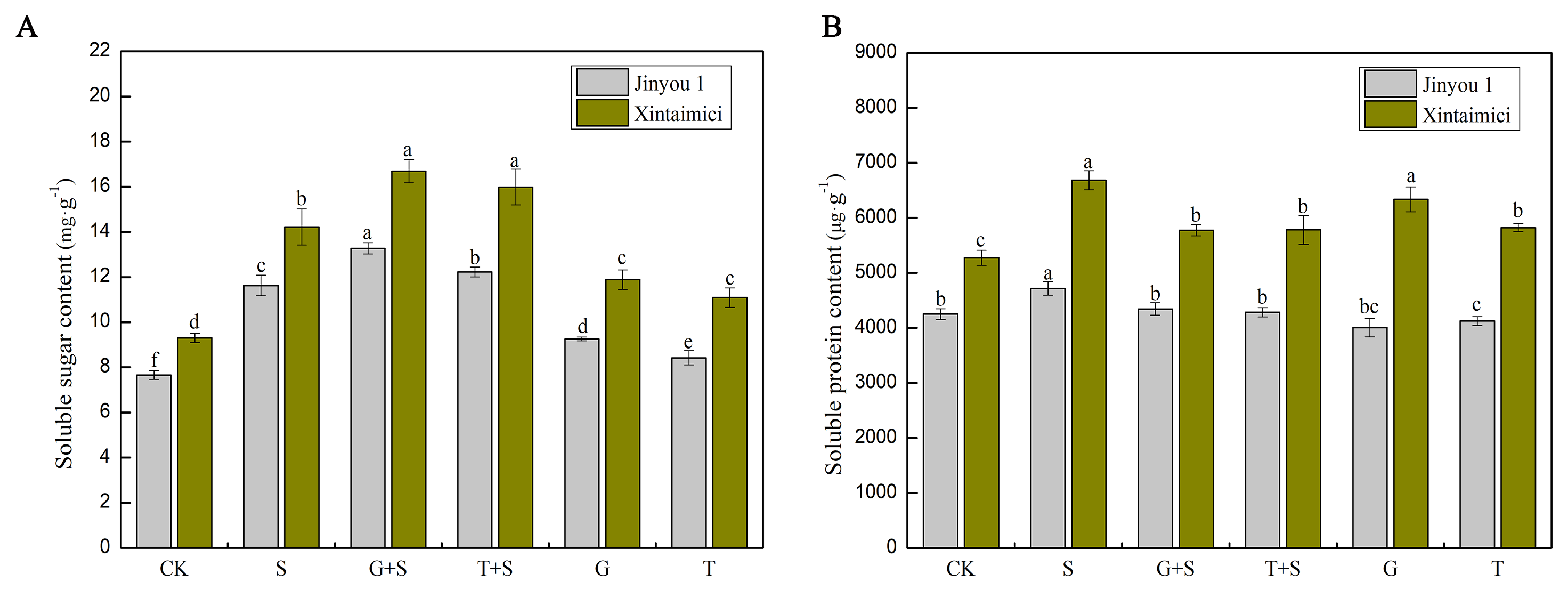

Supplement: Supplementary file 1 [file cimb-47-00754-s001.zip › Figure/Figure 4.tif]

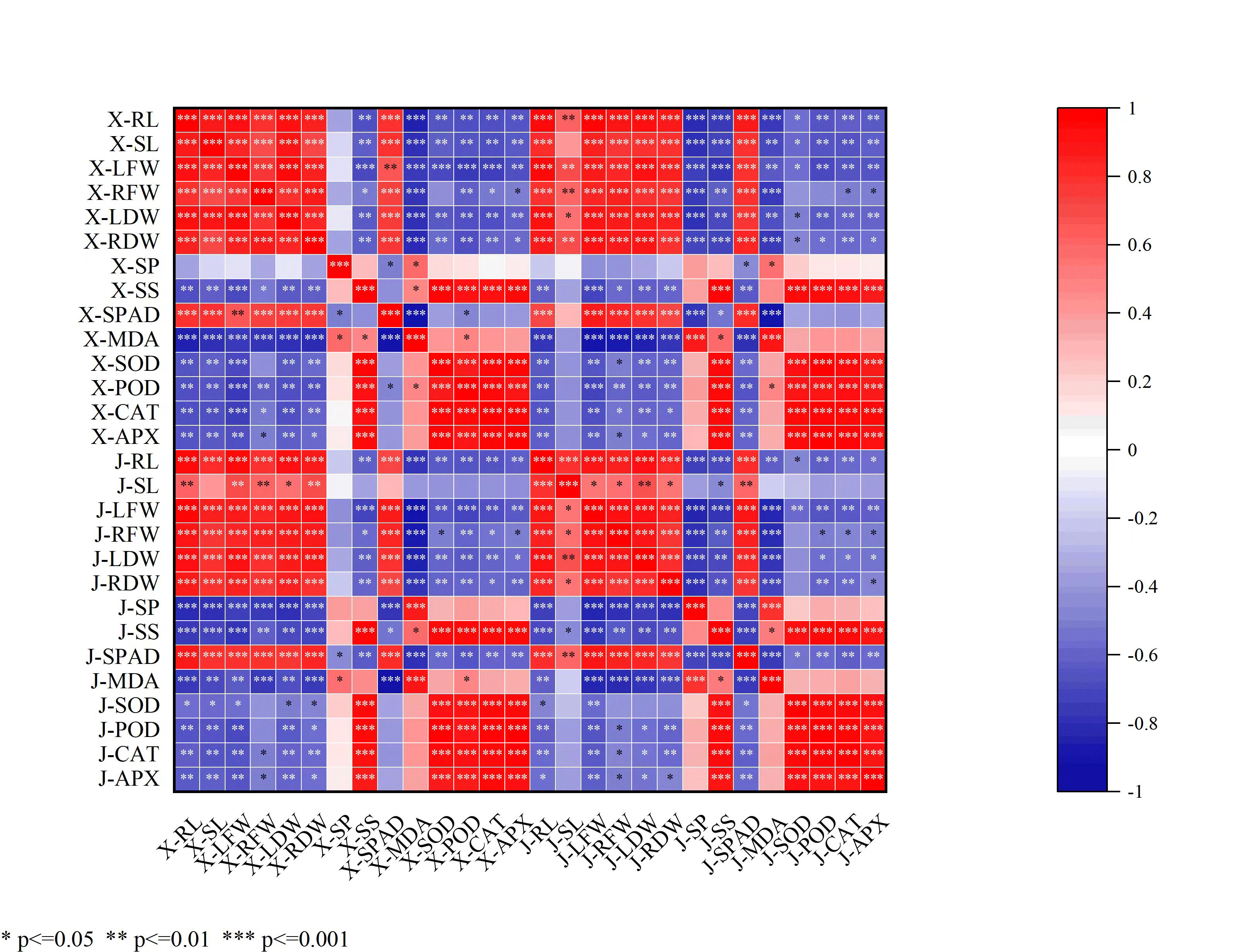

Supplement: Supplementary file 1 [file cimb-47-00754-s001.zip › Figure/Figure 5.tif]

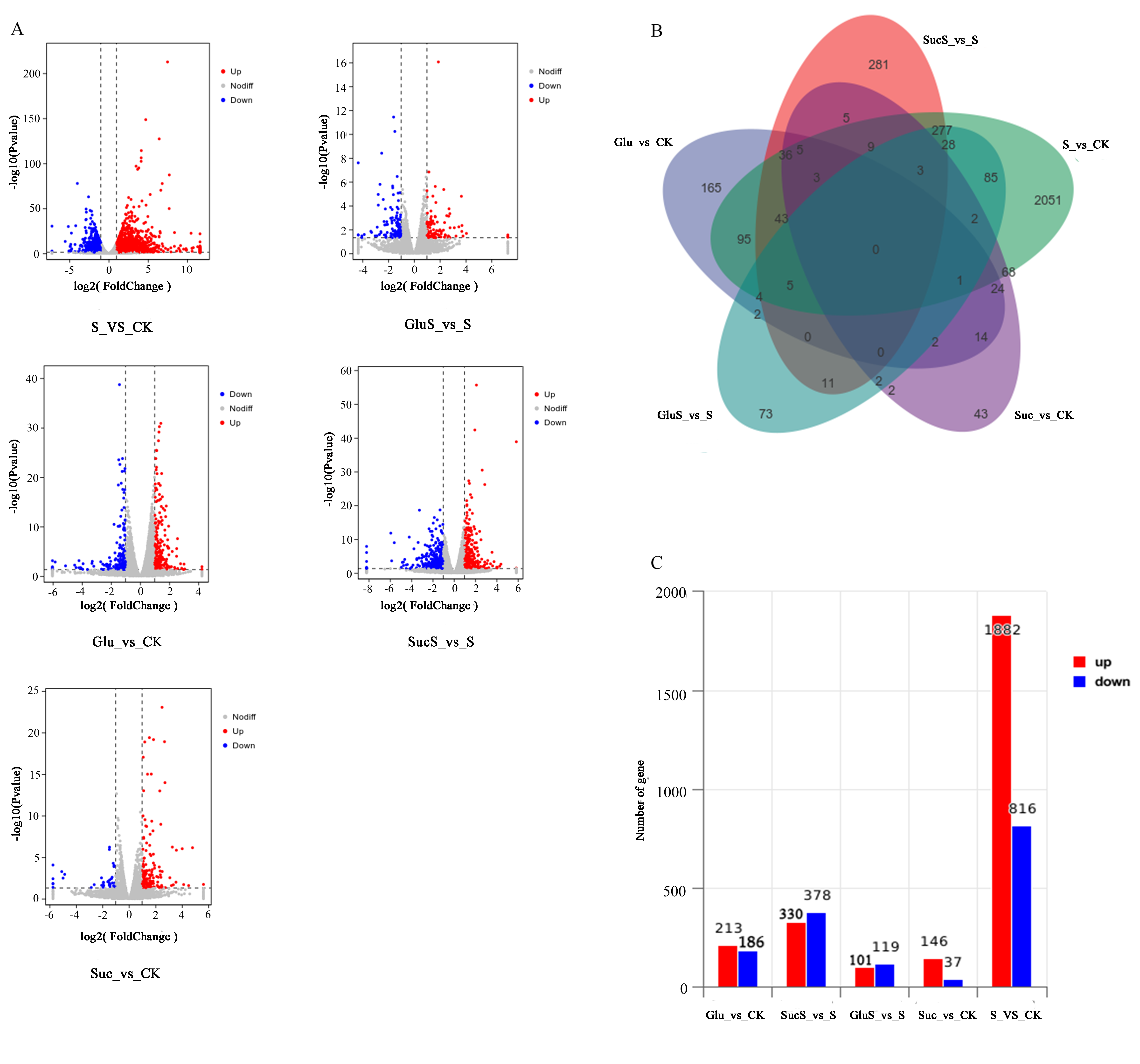

Supplement: Supplementary file 1 [file cimb-47-00754-s001.zip › Figure/Figure 6.tif]

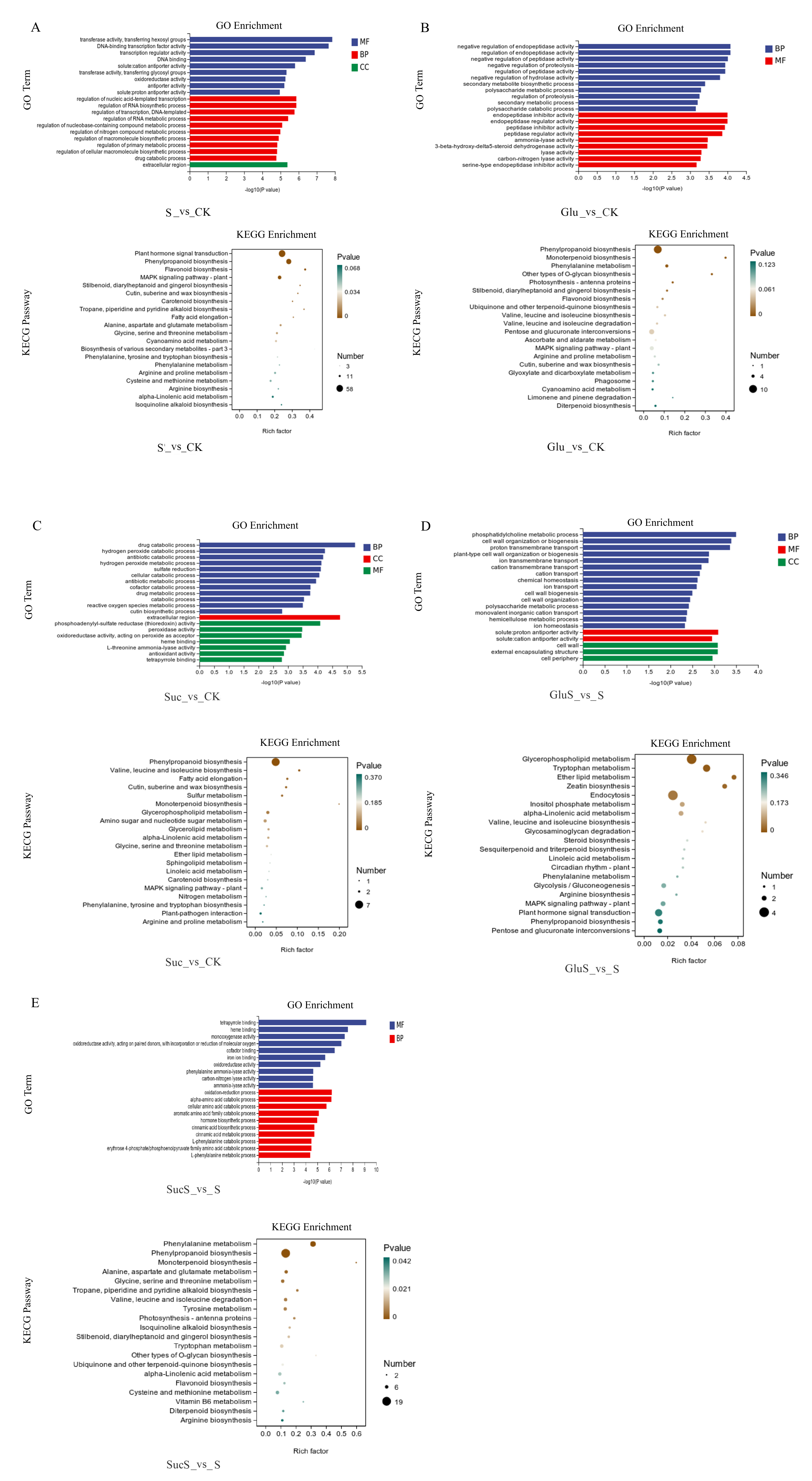

Supplement: Supplementary file 1 [file cimb-47-00754-s001.zip › Figure/Figure 7.tif]

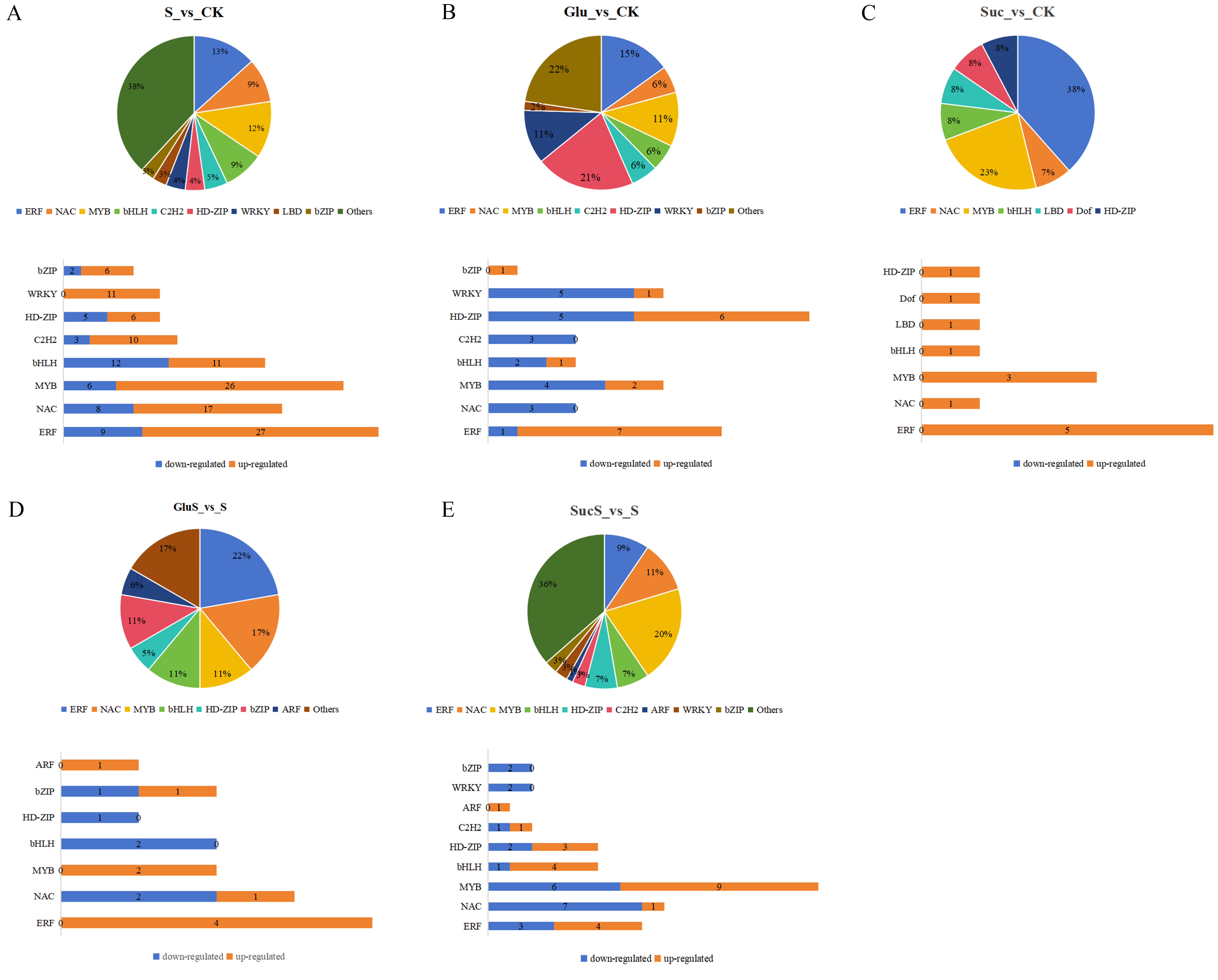

Supplement: Supplementary file 1 [file cimb-47-00754-s001.zip › Figure/Figure 8.tif]
